# Case Report: Immune and Genomic Characteristics Associated With Hyperprogression in a Patient With Metastatic Deficient Mismatch Repair Gastrointestinal Cancer Treated With Anti-PD-1 Antibody

**DOI:** 10.3389/fimmu.2021.749204

**Published:** 2021-09-29

**Authors:** Wenyue Zhou, Yuwen Zhou, Cheng Yi, Xinyao Shu, Guixia Wei, Xiaorong Chen, Xudong Shen, Meng Qiu

**Affiliations:** ^1^ Department of Oncology, West China Hospital, Sichuan University, Chengdu, China; ^2^ The Medical Department, 3D Medicines Inc., Shanghai, China

**Keywords:** MSI-H/dMMR, immune checkpoint inhibitors, hyperprogression, gastrointestinal cancer, case report

## Abstract

Microsatellite instability-high/deficient mismatch repair (MSI-H/dMMR) status of tumors is a distinct predictive biomarker of immune checkpoint inhibitors (ICIs) for colorectal and non-colorectal cancer populations. The overall response rate (ORR) varies from approximately 40% to 60%, indicating that nearly half of MSI-H tumors do not respond to ICIs. The mechanism of response heterogeneity in MSI-H/dMMR cancers is unclear. Some patients who have been treated with ICIs have developed a novel pattern of progression called hyperprogression, which is defined as unexpected accelerated tumor growth. No case of MSI-H/dMMR immunotherapy-associated hyperprogression has been reported in the literature. Here, we present the case of a patient with dMMR gastrointestinal cancer who suffered hyperprogressive disease (HPD) after treatment with nivolumab. We explored the potential mechanisms of HPD by clinical, immune, and genomic characteristics. Extremely high levels of serum LDH, low TMB and TILs, and the disruption of TGFβ signaling, may be related to hyperprogression.

## 1 Introduction

About 2–4% of diagnosed cancer patients are subtyped as “microsatellite instability-high (MSI-H)” or “deficient in mismatch repair (dMMR)” (1) Tumors with these genetic characteristics generally have exceptionally high tumor mutation burden and enriched infiltration of immune cells. These tumors are also known as “hot tumor” (2). Several vital clinical trials have confirmed that the microsatellite instability-high/deficient mismatch repair (MSI-H/dMMR) status is a distinct predictive biomarker of ICIs for colorectal (3) and non-colorectal (1, 4, 5) cancer populations. The overall response rate (ORRs) varies from approximately 40% to 60% (1, 3, 6, 7). Based on the different responses to immunotherapy, this phenomenon attracts significant interest in the heterogeneity and relevant mechanisms of MSI-H/dMMR cancer.

In contrast to chemotherapy and other types of anti-tumor therapy, immunotherapy-induced progression patterns are distinctive, including pseudoprogression and hyperprogression disease (HPD) (8). Pseudoprogression is defined as an initial increase in the tumor burden followed by a later or dissociated response (9). On the other hand, HPD manifests as rapid tumor growth combined with poor overall survival. The acknowledged mechanisms and valuable predictors of HPD remain largely unclear. There is evidence that some gene variations and clinical characteristics may be involved in the mechanism of HPD in different types of cancer. In non-small cell lung cancer and melanoma, patients who harbor MDM2/MDM4 amplification and EGFR alterations are reported to be more likely to develop hyperprogression (10–12). In contrast, those harboring the TP53 mutation without co-existence of STK11 or EGFR mutation have a high CD8 T cell density and, thus, a high response to ICIs (10). Approximately 1–6% of gastrointestinal cancer patients exhibit hyperprogression (HPD) (13). With respect to HPD in MSI-H/dMMR cancers, these phenomena and the mechanisms have not been reported.

Here, we present the first case of a patient with dMMR gastrointestinal cancer with primary resistance to immunotherapy, who experienced HPD after two doses of nivolumab. Nivolumab is a programmed death receptor-1 (PD-1)-blocking antibody and is approved for different types of cancer. Rapid hyperprogression was not observed. Pre-immunotherapy circulating tumor DNA (ctDNA) analysis using 150-gene panel next-generation sequencing, immunohistochemistry, multiplex fluorescence immunohistochemistry (mIHC), and blood inflammatory indexes were used to examine the immune microenvironment as well as the patient’s genomic and clinical features. We also explored possible mechanisms of HPD. Furthermore, we identified the underlying mechanism and demonstrated the importance of personalized treatment for clinical practice.

## 2 Case Description

### 2.1 Case Presentation

**Figure 1:**
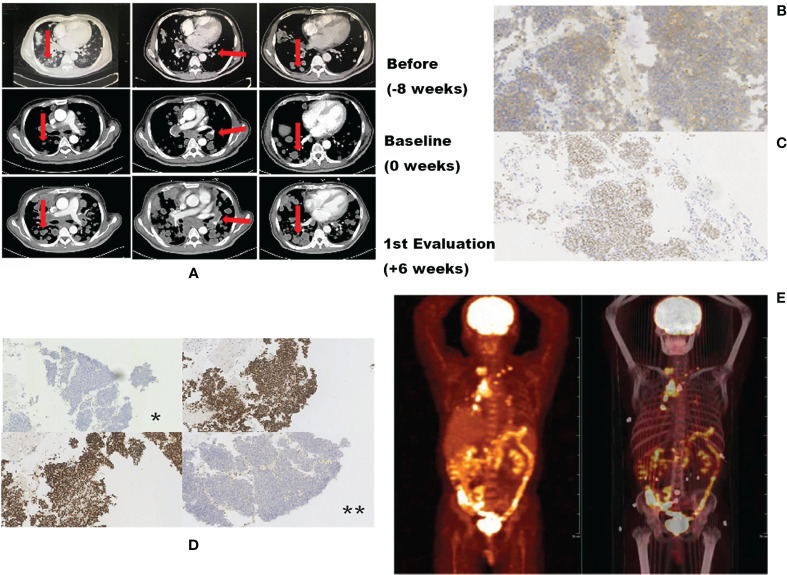
Radiologic images before and after nivolumab showing distinct TGR differences. **(A)** Representative computed tomography scans of the patient’s lung before, at baseline, and during treatment. **(B)** Second immunohistochemistry (IHC) showing CDX2 positivity, indicating that the possible original tumor site may be the gastrointestinal duct. **(C)** Second IHC indicating a PD-L1 expression rate of 5–10%. **(D)** Second IHC showing loss of MMR protein expression; *loss of MLH1 protein expression; **loss of expression of PMS2 protein. **(E)** Positron emission tomography/computed tomography (PET-CT) scan suggesting extensive tumor burden.

A 47-year-old male patient presented with constant abdominal pain for 4 months and was sent to the hospital for physical examination in October 2017. Computed tomography (CT) revealed multiple masses in the pancreatic body and both the lungs, with enlarged abdominal and supraclavicular lymph nodes, suggesting a heavy tumor load. The patient underwent supraclavicular lymph node dissection. Immunohistochemistry results suggested poorly differentiated metastatic adenocarcinoma. Based on CT imaging, the doctor-in-charge diagnosed that the tumor originated from the pancreas. Gemcitabine plus paclitaxel (Abraxane) chemotherapy was used as first-line treatment. Two months later, follow-up CT revealed tumor progression in both the lungs (**Figure 1A**). Positron-emission tomography–CT revealed increased intake of ^18^F-fluorodeoxyglucose in the intestinal mesentery, cervicothoracic, abdominal lymph nodes, and both the lungs, indicating significant tumor load once again (**Figure 1D**). One enlarged abnormal cervical lymph node was punctured for examination because the patient refused dissection biopsy and immunohistochemistry was used to analyze the possible site of origin, tumor expression of MMR protein, and gene variation. Immunohistochemistry (IHC) of the second biopsy indicated that the tumor most likely originated from the gastrointestinal duct (IHC showed CDX2 positivity as shown in **Figure 1B**, microscopic magnification ×100). A 5–10% loss of MLH1 and PMS2 protein expression and PD-L1 expression was also observed (**Figures 1C, D**, microscopic magnification ×100), suggesting a dMMR status in this patient. With a performance status (PS) score of 1, nivolumab (200 mg every 2 weeks) was used as second-line treatment. Unfortunately, 1.5 month after the commencement of nivolumab (one dose of nivolumab), the patient experienced rapid deterioration in lung function, including obvious dyspnea and lethargy, and his performance status score deteriorated to 3. A follow-up chest CT scan confirmed rapid metastatic progression in the lungs and lymph nodes (**Figure 1A**). Moreover, laboratory tests revealed significant high serum lactate dehydrogenase (1360 IU/L, ranging from to 110-220 IU/L). Two days later, the patient died of respiratory failure.

### 2.2 Diagnostic Assessments

#### 2.2.1 Blood Test and Biopsy

Serum lactate dehydrogenase and other blood tests were obtained the day before each treatment. The dissected lymph supraclavicular lymph node, and punctured cervical lymph node tissues were obtained under ultrasound and CT guidance, respectively.

#### 2.2.2 Diagnosis of HPD

Currently, the most accepted definition of HPD is based on the study by Baptiste et al. (9) which detailed the following criteria: time to failure <2 months and TGK_exp_: TGK_ref_ >2. Tumor growth kinetics (TGK) are estimated to quantitatively assess the progression of the patient. According to Baptiste’s definition, our patient’s TGK_exp_ and TGK_ref_ were 2.1 and 0.7.8, respectively, and the ratio (TGK_exp_: TGK_ref_) was 2.18. This value was calculated with reference to a single lesion, but the CT scan revealed a large number of newly developed lesions (**Figure 1A**). The tumor growth curve as shown in **Figure 1E** also indicated explosive tumor progression. The time to failure of the patient was 1.5 months. The ratio of TGK_exp_ and TGK_ref_ was 2.18. As such, he met the definition of immunotherapy hyperprogression.

#### 2.2.3 Gene Analysis

**Figure 2:**
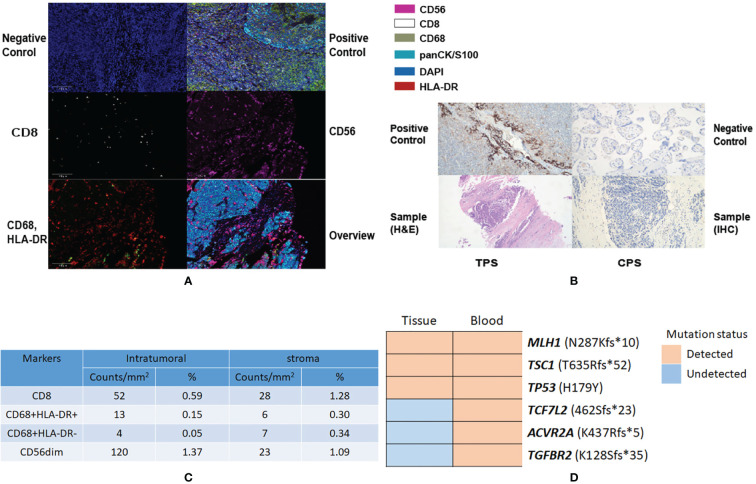
Results showing tumor microenvironment and genomic mutation copies alteration in autopsy specimens. **(A)** Representative images of PanCK (pan cytokeratin), CD56, CD8, CD68, and HLA-DR as shown by mIHC. Nuclei (blue) and counterstained by DAPI. Original magnification ×200. **(B)** Tumor proportion score (TPS) and combined positive score (CPS). **(C)** Quantification results of TIL percentages by mIHC in the tumor stroma and margin. **(D)** Alteration of the genomic mutation copies before and after immunotherapy.

A 150-gene panel next generation sequencing (NGS) of circulating tumor DNA analysis (3D Medicines Co., Shanghai) before administration of nivolumab revealed six gene mutations with high frequency, including MLH1 p.N287Kfs (56.32%), TCF7L2 p.K462Sfs (36.85%), TP53 p.H179Y (41.4%), ACVR2A p.K437Rfs (69.51%), TGFBR2 p.K128Sfs (66.46%), and TSC1 p.T635Rfs (5.41%). Tumor NGS was detected *via* the punctured tissues. Three mutation genes were inspected (**Figure 2D**), including MLH1 p.N287Kfs (34.3%), TP53 p.H179Y (38.4%), and TSC1 p.T635Rfs (1.7%). Tumor mutation burden (TMB) was also calculated. With a TMB of 7.26 Muts/Mb, the tumor was categorized as low TMB.

#### 2.2.4 Analysis of the Tumor Immune Microenvironment

To further explore the possible refractory mechanism of HPD in this patient, the lymph node tissue tumor microenvironment (TME) of the patient was examined and PD-L1 protein expression was detected in the formalin-fixed, paraffin-embedded (FFPE) punctured tumor tissues using the Dako PD-L1 IHC 22C3 pharmDx assay. The expression levels of CD8+, CD68+, CD56+ cells, pan cytokeratin, and HLA-DR were assessed and calculated using multiplex fluorescence immunohistochemistry (mIHC). Quantitative analysis of the tumor microenvironment showed that the degree of CD8+, CD68+, and CD56+ cells in the tumor stroma, and tumor region, were 0.59% and 1.28%, respectively, along with 0.15%, 0.30%, and 0.63% and 0.49%, respectively (**Figure 2C**).

## 3 Discussion

This is the first reported case of HPD in MSI-H/dMMR gastrointestinal cancer, with no HPD-related gene mutation found in our patient. This is also the first report to demonstrate characteristics such as performance status, laboratory tests, TMB, TME, and tumor immune infiltrations (TILs) factors, and some gene alterations, to explain the case better.

Some clinical parameters are reliable predictive prognostic factors for patients with ICI-treated cancer. Studies have shown that high levels of serum lactate dehydrogenase (LDH), tumor burden, and poor performance status are independent baseline factors associated with hyperprogression (14, 15). According to the authors, LDH levels are strongly correlated with prognosis. The prognosis of patients within the normal range is within 16.1 months, but decreases to 2.3 months for those with LDH levels 2.5 times above the upper limit. The authors also demonstrated that low tumor burden and good performance status are also characteristics of favorable OS. As described, the patient had poor performance status, high LDH level, and a rather high tumor burden. The unfortunate outcome was consistent with previous conclusions.

In this case, the patient went through HPD although his TMB was 7.26 Muts/Mb (150-gene panel). TMB is stratified into two subtypes: low and high TMB. High TMB is usually defined as above the median burden based on whole exome sequencing (WES) or different gene panels (16). Optimal TMB cut-offs vary among different studies. However, levels lower than 10 Muts/Mb are generally categorized as low (1, 11, 17). TMB is a reliable efficacy factor for immunotherapy (11, 18–21). Studies have demonstrated that those with low TMB, as seen in this case, showed less favorable survival rate (11, 17, 18, 20–24). MSI-H/dMMR tumors are identified with high clonality of non-synonymous mutations (also high TMB), leading to the presence of robust altered proteins, known as neoantigens (25). Neoantigens are thought to be relevant to tumor control, as they can be targeted by the immune system, leading to a series of cytolytic activities of the immune cells (1, 3, 25–27). With ORRs varies from 31.7% to 40% (3, 28, 29), nivolumab was approved for the treatment of melanoma, pancreatic, colorectal cancer, gastric cancer and so on. The intolerance and toxicities are mostly immune-mediated, along with infusion-related reactions and embryo-fatal adverse events. In this case, it is not hard to speculate that the low TMB reflected a minimal neoantigen, and was thus refractory to nivolumab. Hence, it is reasonable to assume that the heterogeneity of dMMR is partially responsible for the low levels of TMB and hyperprogression.

Heterogeneity of MSI-H/dMMR TILs was also observed by Harry H. Yoon et al. (30) TILs have been considered as positive prognostic predictive factors in tumor patients since the early 1900s. It is widely acknowledged that CD8+ TIL percentage is the most important predictive factor in immunotherapy (31). Compared with the MSS/pMMR status, CD3+ and CD8+ TILs are significantly more abundant in MSI-H/dMMR patients (30). In this case, as shown in **Figures 2A, C**, CD8+ lymphocytes, CD68+ macrophages, and CD56+ NK cell infiltration percentages were rather low. The PD-L1 assay (**Figure 2A**, original magnification ×200) also revealed extremely low lymphocytes. According to Yoon et al., lower TILs in dMMR are associated with younger, non-smoking, and low histological grade patients. The tumor proportion score (TPS) and combined positive score (CPS) were also examined (**Figure 2B**, original magnification ×200). The patient’s TPS and CPS were 0% and 0%, respectively (Dako 22C3 pharmDx, Santa Barbara, CA).

TME is classified into four types based on T cell infiltration and the presence of PD-L1 (32), and TME type III was categorized in this case. While T cell infiltration was observed in the patient, it appears that some unknown mechanisms prevented the effector T cells from functioning normally. A recent study also described that abnormal function of effector T cells may be a hindrance to their proliferation, execution, and maturation in immunotherapy-resistant TME type III patients (33). In addition, TILs in the tumor context have been proven to be a major positive prognostic predictive factor (34). Hence, low TIL percentages in patients cannot be neglected when attempting to elucidate the mechanism. Finally, although not yet listed as a negative factor of immunotherapy, some studies have indicated that TGFβ signaling could restrict T cells movement in TM. This then leads to a lack of response to anti-PD therapy and hyperprogression (35, 36).

Recent studies of TGFβ signaling may elucidate the possible mechanisms of hyperprogression. TGFβ is a bifunctional regulator that plays an important role in tumor initiation and progression, and deletions in the TGFBR2 gene loci can lead to TGFβ signaling disruption. Studies have confirmed that TGFβ-derived epithelial-mesenchymal transition (EMT) can increase mesenchymal cells and lead to tissue fibrosis (34, 37). The pivotal role of TGFβ in tumor microenvironment (TME) fibrogenesis suggests that TGFβ signaling may restrict T cell movement and counteract anti-tumor immunity (35). TGFβ also plays a vital role in immunotherapy. By limiting the infiltration of inflammatory/immune cells, it could suppress CD8+ T cells and NK cell-mediated anti-tumor response (38). TGFβ inhibits T cell proliferation at the transcriptional level and represses their cytotoxic activity, resulting in tumor progression, and in some cases, hyperprogression. Xiong et al. found regulated TGFβ in the consistent sequencing of two patients with HPD (36). Unfortunately, our patient did not exhibit such a tendency (**Figure 2D**), but the small sample size in the study by Xiong et al. limited its credibility. Although TGFβ has not yet been confirmed as a negative regulator in immunotherapy, it is a new mechanism that merits further exploration. Apart from TGFβ, deregulated WNT signaling could also increase resistance to TILs, as long as there is activation of epithelial-to-mesenchymal transition (EMT), and both methods could limit the infiltration of TILs, especially CD8+ lymph cells (39). In conclusion, TGFβ activation and WNT deregulation may explain the reason for nivolumab resistance and hyperprogression in this case.

Thus, HPD is a comprehensive-cause phenomenon. Studies have suggested that poor clinical characteristics, such as low TMB, heterogeneity of MSI-H/dMMR status, low levels of TILs, TGFBR2 alteration, and TME type III patients could develop mechanism-unknown immune resistance. In this case, they may also lead to HPD.

## Data Availability Statement

The original contributions presented in the study are publicly available. This data can be found here: https://www.ncbi.nlm.nih.gov/sra/PRJNA756873.

## Ethics Statement

This study was approved by the institutional review board of the West China Medical College of Sichuan University (ethical approval letter 20211077). The patient provided informed consent for his treatments, the collection and study of his blood and tissue samples. All procedures were conducted according to the Declaration of Helsinki. Written informed consent was obtained from the participant for the publication of this case report.

## Author Contributions

MQ conceived the idea of the article. WZ mainly takes charge of writing and figure-making. YZ and CY plays a guiding role in article revision, polish, genetic analysis, and ethical review. All authors contributed to the revision and discussion. All authors contributed to the article and approved the submitted version.

## Conflict of Interest

XDS is employed by 3D Medicines Inc.

The remaining authors declare that the research was conducted in the absence of any commercial or financial relationships that could be construed as a potential conflict of interest.

## Publisher’s Note

All claims expressed in this article are solely those of the authors and do not necessarily represent those of their affiliated organizations, or those of the publisher, the editors and the reviewers. Any product that may be evaluated in this article, or claim that may be made by its manufacturer, is not guaranteed or endorsed by the publisher.

## References

[1] Marabelle A Le DT Ascierto PA Di Giacomo AM Jesus-Acosta A D Delord J-P. Efficacy of Pembrolizumab in Patients With Noncolorectal High Microsatellite Instability or Mismatch Repair-Deficient Cancer, Results From the Phase II KEYNOTE-158 Study. J Clin Oncol (2019) 37:1–10. doi: 10.1200/JCO.19.02105 31682550 PMC8184060

[2] Galon J Bruni D. Approaches to Treat Immune Hot, Altered and Cold Tumours With Combination Immunotherapies. Nat Rev Drug Discov (2019) 18(3):197–218. doi: 10.1038/s41573-018-0007-y 30610226

[3] Overman MJ McDermott R Leach JL Lonardi S Lenz H-J Morse MA. Nivolumab in Patients With Metastatic DNA Mismatch Repair-Deficient or Microsatellite Instability-High Colorectal Cancer (CheckMate 142): An Open-Label, Multicentre, Phase 2 Study. Lancet Oncol (2017) 18(9):1182–91. doi: 10.1016/S1470-2045(17)30422-9 PMC6207072 28734759

[4] Smith KN Le DT Durham JN. Mismatch-Repair Deficiency Predicts Response of Solid Tumors to PD-1 Blockade. Science (2017) 357:409–13. doi: 10.1126/science.aan6733 PMC5576142 28596308

[5] Le DT Uram JN Wang H Bartlett BR Kemberling H Eyring AD. PD-1 Blockade in Tumors With Mismatch-Repair Deficiency. N Engl J Med (2015) 372(26):2509–20. doi: 10.1056/NEJMoa1500596 PMC4481136 26028255

[6] Muro K Chung HC Shankaran V Geva R Catenacci D Gupta S. Pembrolizumab for Patients With PD-L1-Positive Advanced Gastric Cancer (KEYNOTE-012): A Multicentre, Open-Label, Phase 1b Trial. Lancet Oncol (2016) 17(6):717–26. doi: 10.1016/S1470-2045(16)00175-3 27157491

[7] Le DT Kim TW Cutsem EV Geva R Jäger D Hara H. Phase II Open-Label Study of Pembrolizumab in Treatment-Refractory, Microsatellite Instability-High/Mismatch Repair-Deficient Metastatic Colorectal Cancer: KEYNOTE-164. J Clin Oncol (2019) 38:11–9. doi: 10.1200/JCO.19.02107 PMC7031958 31725351

[8] Fuentes-Antras J Provencio M Diaz-Rubio E. Hyperprogression as a Distinct Outcome After Immunotherapy. Cancer Treat Rev (2018) 70:16–21. doi: 10.1016/j.ctrv.2018.07.006 30053725

[9] Kas B Talbot H Ferrara R Richard C Lamarque JP Pitre-Champagnat S. Clarification of Definitions of Hyperprogressive Disease During Immunotherapy for Non-Small Cell Lung Cancer. JAMA Oncol (2020) 6(7):1039–46. doi: 10.1001/jamaoncol.2020.1634 PMC7290708 32525513

[10] Biton J Mansuet-Lupo A Pecuchet N Alifano M Ouakrim H Arrondeau J. TP53, STK11, and EGFR Mutations Predict Tumor Immune Profile and the Response to Anti-PD-1 in Lung Adenocarcinoma. Clin Cancer Res (2018) 24(22):5710–23. doi: 10.1158/1078-0432.CCR-18-0163 29764856

[11] Samstein RM Lee CH Shoushtari AN Hellmann MD Shen R Janjigian YY. Tumor Mutational Load Predicts Survival After Immunotherapy Across Multiple Cancer Types. Nat Genet (2019) 51(2):202–6. doi: 10.1038/s41588-018-0312-8 PMC6365097 30643254

[12] Gatalica Z Xiu J Swensen J Vranic S. Comprehensive Analysis of Cancers of Unknown Primary for the Biomarkers of Response to Immune Checkpoint Blockade Therapy. Eur J Cancer (2018) 94:179–86. doi: 10.1016/j.ejca.2018.02.021 29571084

[13] Champiat S Dercle L Ammari S Massard C Hollebecque A Postel-Vinay S. Hyperprogressive Disease Is a New Pattern of Progression in Cancer Patients Treated by Anti-PD-1/PD-L1. Clin Cancer Res (2017) 23(8):1920–8. doi: 10.1158/1078-0432.CCR-16-1741 27827313

[14] Weide B Martens A Hassel JC Berking C Postow MA Bisschop K. Baseline Biomarkers for Outcome of Melanoma Patients Treated With Pembrolizumab. Clin Cancer Res (2016) 22(22):5487–96. doi: 10.1158/1078-0432.CCR-16-0127 PMC5572569 27185375

[15] Kim JY Lee KH Kang J Borcoman E Saada-Bouzid E Kronbichler A. Hyperprogressive Disease During Anti-PD-1 (PDCD1)/PD-L1 (CD274) Therapy: A Systematic Review and Meta-Analysis. Cancers (Basel) (2019) 11(11):1699. doi: 10.3390/cancers11111699 PMC6896059 31683809

[16] Rizvi NA Hellmann MD Snyder A Kvistborg P Makarov V Havel JJ. Mutational Landscape Determines Sensitivity to PD-1 Blockade in Non–Small Cell Lung Cancer. Science (2016) 348(6230):124–8. doi: 10.1126/science.aaa1348 PMC4993154 25765070

[17] Chida K Kawazoe A Kawazu M Suzuki T Nakamura Y Nakatsura T. A Low Tumor Mutational Burden and PTEN Mutations are Predictors of a Negative Response to PD-1 Blockade in MSI-H/dMMR Gastrointestinal Tumors. Clin Cancer Res (2021) 27:3714–24. doi: 10.1158/1078-0432.CCR-21-0401 33926917

[18] Leslie M. High TMB Predicts Immunotherapy Benefit. Cancer Discov (2018) 8(6):668. doi: 10.1158/2159-8290.CD-NB2018-048 29661758

[19] Saâda-Bouzid E Defaucheux C Karabajakian A Coloma VP Servois V Paoletti X. Hyperprogression During Anti-PD-1/PD-L1 Therapy in Patients With Recurrent and/or Metastatic Head and Neck Squamous Cell Carcinoma. Ann Oncol (2017) 28(7):1605–1611. doi: 10.1093/annonc/mdx178 28419181

[20] Cristescu R Mogg R Ayers M Albright A Murphy E Yearley J. Pan-Tumor Genomic Biomarkers for PD-1 Checkpoint Blockade-Based Immunotherapy. Science (2018) 362(6411):eaar3593. doi: 10.1126/science.aar3593 30309915 PMC6718162

[21] Jones S Anagnostou V Lytle K Parpart-Li S Nesselbush M Riley DR. Personalized Genomic Analyses for Cancer Mutation Discovery and Interpretation. Cancer (2015) 7(283):283ra53. doi: 10.1126/scitranslmed.aaa7161 PMC4442685 25877891

[22] Liu L Bai X Wang J Tang XR Wu DH Du SS. Combination of TMB and CNA Stratifies Prognostic and Predictive Responses to Immunotherapy Across Metastatic Cancer. Clin Cancer Res (2019) 25(24):7413–23. doi: 10.1158/1078-0432.CCR-19-0558 31515453

[23] Petrelli F Ghidini M Ghidini A Tomasello G. Outcomes Following Immune Checkpoint Inhibitor Treatment of Patients With Microsatellite Instability-High Cancers: A Systematic Review and Meta-Analysis. JAMA Oncol (2020) 6(7):1068–71. doi: 10.1001/jamaoncol.2020.1046 PMC7226284 32407439

[24] Lu S Stein JE Rimm DL Wang DW Bell JM Johnson DB. Comparison of Biomarker Modalities for Predicting Response to PD-1/PD-L1 Checkpoint Blockade: A Systematic Review and Meta-Analysis. JAMA Oncol (2019) 5(8):1195–204. doi: 10.1001/jamaoncol.2019.1549 PMC6646995 31318407

[25] Baretti M Le DT. DNA Mismatch Repair in Cancer. Pharmacol Ther (2018) 189:45–62. doi: 10.1016/j.pharmthera.2018.04.004 29669262

[26] Yang W Lee KW Srivastava RM Kuo F Krishna C Chowell D. Immunogenic Neoantigens Derived From Gene Fusions Stimulate T Cell Responses. Nat Med (2019) 25(5):767–75. doi: 10.1038/s41591-019-0434-2 PMC6558662 31011208

[27] Schumacher TN Schreiber RD. Neoantigens in Cancer Immunotherapy. Science (2015) 348(6230):69–74. doi: 10.1126/science.aaa4971 25838375

[28] Weber JS D’Angelo SP Minor D Hodi FS Gutzmer R Neyns B. Nivolumab Versus Chemotherapy in Patients With Advanced Melanoma Who Progressed After Anti-CTLA-4 Treatment (CheckMate 037): A Randomised, Controlled, Open-Label, Phase 3 Trial. Lancet Oncol (2015) 16(4):375–84. doi: 10.1016/S1470-2045(15)70076-8 25795410

[29] Robert C Long GV Brady B Dutriaux C Maio M Mortier L. Nivolumab in Previously Untreated Melanoma Without BRAF Mutation. N Engl J Med (2015) 372(4):320–30. doi: 10.1056/NEJMoa1412082 25399552

[30] Yoon HH Shi Q Heying EN Muranyi A Bredno J Ough F. Intertumoral Heterogeneity of CD3(+) and CD8(+) T-Cell Densities in the Microenvironment of DNA Mismatch-Repair-Deficient Colon Cancers: Implications for Prognosis. Clin Cancer Res (2019) 25(1):125–33. doi: 10.1158/1078-0432.CCR-18-1984 PMC6320300 30301825

[31] Chiou SH Sheu BC Chang WC Huang SC Hong-Nerng H. Current Concepts of Tumor-Infiltrating Lymphocytes in Human Malignancies. J Reprod Immunol (2005) 67(1-2):35–50. doi: 10.1016/j.jri.2005.06.002 16111767

[32] Taube JM Anders RA Young GD Xu H Sharma R McMiller TL. Colocalization of Inflammatory Response With B7-H1 Expression in Human Melanocytic Lesions Supports an Adaptive Resistance Mechanism of Immune Escape. Sci Trans Med (2012) 4(127):127ra37. doi: 10.1126/scitranslmed.3003689 PMC3568523 22461641

[33] Kim TK Herbst RS Chen L. Defining and Understanding Adaptive Resistance in Cancer Immunotherapy. Trends Immunol (2018) 39(8):624–31. doi: 10.1016/j.it.2018.05.001 PMC6066429 29802087

[34] Bremnes RM Busund L-T Kilvær TL Andersen S Richardsen E Paulsen EE. The Role of Tumor-Infiltrating Lymphocytes in Development, Progression, and Prognosis of Non–Small Cell Lung Cancer. J Thorac Oncol (2016) 11(6):789–800. doi: 10.1016/j.jtho.2016.01.015 26845192

[35] Mariathasan S Turley SJ Nickles D Castiglioni A Yuen K Wang Y. TGFbeta Attenuates Tumour Response to PD-L1 Blockade by Contributing to Exclusion of T Cells. Nature (2018) 554(7693):544–8. doi: 10.1038/nature25501 PMC6028240 29443960

[36] Xiong D Wang Y Singavi AK Mackinnon AC George B You M. Immunogenomic Landscape Contributes to Hyperprogressive Disease After Anti-PD-1 Immunotherapy for Cancer. iScience (2018) 9:258–77. doi: 10.1016/j.isci.2018.10.021 PMC6234258 30439581

[37] Morikawa M Derynck R Miyazono K. TGF-Beta and the TGF-Beta Family: Context-Dependent Roles in Cell and Tissue Physiology. Cold Spring Harb Perspect Biol (2016) 8(5):a021873. doi: 10.1101/cshperspect.a021873 27141051 PMC4852809

[38] Yang L Pang Y Moses HL. TGF-Beta and Immune Cells: An Important Regulatory Axis in the Tumor Microenvironment and Progression. Trends Immunol (2010) 31(6):220–7. doi: 10.1016/j.it.2010.04.002 PMC2891151 20538542

[39] Galluzzi L Spranger S Fuchs E Lopez-Soto A. WNT Signaling in Cancer Immunosurveillance. Trends Cell Biol (2019) 29(1):44–65. doi: 10.1016/j.tcb.2018.08.005 30220580 PMC7001864

